# Manipulating magnetoelectric energy landscape in multiferroics

**DOI:** 10.1038/s41467-020-16727-2

**Published:** 2020-06-05

**Authors:** Yen-Lin Huang, Dmitri Nikonov, Christopher Addiego, Rajesh V. Chopdekar, Bhagwati Prasad, Lei Zhang, Jyotirmoy Chatterjee, Heng-Jui Liu, Alan Farhan, Ying-Hao Chu, Mengmeng Yang, Maya Ramesh, Zi Qiang Qiu, Bryan D. Huey, Chia-Ching Lin, Tanay Gosavi, Jorge Íñiguez, Jeffrey Bokor, Xiaoqing Pan, Ian Young, Lane W. Martin, Ramamoorthy Ramesh

**Affiliations:** 1 Department of Materials Science and Engineering, University of California, Berkeley, Berkeley, CA 94720 USA; 2 Materials Sciences Division, Lawrence Berkeley Laboratory, Berkeley, CA 94720 USA; 3 Exploratory Integrated Circuits, Components Research, Intel Corp., Hillsboro, Oregon 97124 USA; 4 Department of Physics and Astronomy, University of California, Irvine, Irvine, CA 92697 USA; 5 Advanced Light Source, Lawrence Berkeley National Laboratory, Berkeley, CA 94720 USA; 6 Electrical Engineering and Computer Sciences, University of California, Berkeley, Berkeley, CA 94720 USA; 7 Department of Materials Science and Engineering, National Chung Hsing University, Taichung, 402 Taiwan; 8 Department of Applied Physics, Aalto University School of Science, Espoo, FI-00076 Finland; 9 Department of Materials Science and Engineering, National Chaio Tung University, Hsinchu, 300 Taiwan; 10 Department of Physics, University of California, Berkeley, Berkeley, CA 94720 USA; 11 Department of Physics, University of California, Irvine, Santa Cruz, CA 95064 USA; 12 Department of Materials Science and Engineering, University of Connecticut, Storrs, CT 06269 USA; 13 Materials Research and Technology Department, Luxembourg Institute of Science and Technology (LIST), 5 avenue des Hauts-Fourneaux, L-4362 Esch/Alzette, Luxemburg; 14 Physics and Materials Science Research Unit, University of Luxembourg, 41, rue du Brill, L-4422 Belvaux, Luxembourg; 15 Department of Materials Science and Engineering, University of California, Irvine, Irvine, CA 92697 USA; 16 Irvine Materials Research Institute, University of California, Irvine, Irvine, CA 92697 USA

**Keywords:** Condensed-matter physics, Nanoscale materials

## Abstract

Magnetoelectric coupling at room temperature in multiferroic materials, such as BiFeO_3_, is one of the leading candidates to develop low-power spintronics and emerging memory technologies. Although extensive research activity has been devoted recently to exploring the physical properties, especially focusing on ferroelectricity and antiferromagnetism in chemically modified BiFeO_3_, a concrete understanding of the magnetoelectric coupling is yet to be fulfilled. We have discovered that La substitutions at the Bi-site lead to a progressive increase in the degeneracy of the potential energy landscape of the BiFeO_3_ system exemplified by a rotation of the polar axis away from the 〈111〉_pc_ towards the 〈112〉_pc_ discretion. This is accompanied by corresponding rotation of the antiferromagnetic axis as well, thus maintaining the right-handed vectorial relationship between ferroelectric polarization, antiferromagnetic vector and the Dzyaloshinskii-Moriya vector. As a consequence, La-BiFeO_3_ films exhibit a magnetoelectric coupling that is distinctly different from the undoped BiFeO_3_ films.

## Abstract (web summary)

BiFeO_3_ has a wide application but the impact of rare-earth substitution for the evolution of the coupling mechanism is unknown. Here, the authors reveal the correlation between ferroelectricity, antiferromagnetism, a weak ferromagnetic moment, and their switching pathways in La-substituted BiFeO_3_.

## Introduction

**Fig. 1:**
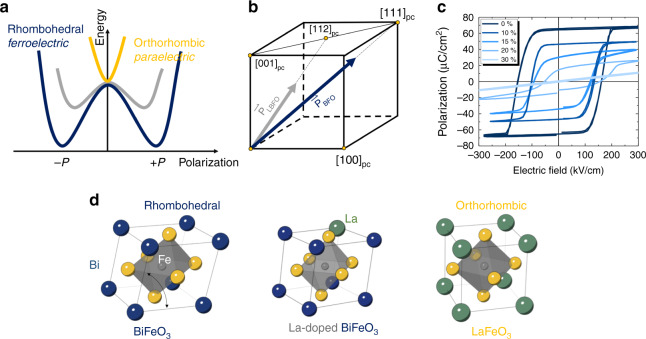
Ferroelectric ordering in Bi_1−*x*_La_*x*_FeO_3_. **a** Schematic for the energy landscape of the phase transition induced by lanthanum substitution in BiFeO_3_ described by Landau theory. **b** Schematic for the ferroelectric polarization rotation (from BiFeO_3_: [111]_pc_ to Bi_0.85_La_0.15_FeO_3_: [112]_pc_) and suppression of ferroelectric polarization induced by lanthanum substitution in BiFeO_3_. **c** *P-E* measurements for different substitution levels of lanthanum in 100-nm-thick BiFeO_3_ films. **d** The schematics illustrate the evolution of crystal symmetry of bismuth ferrite (rhombohedral) to lanthanum ferrite (orthorhombic).

Magnetoelectric multiferroics are materials which possess two or more order parameters simultaneously and, more importantly, exhibit coupling between the spin and charge degrees of freedom^1,2^. Bismuth ferrite (BiFeO_3_) is by far the most studied and characterized multiferroic in part because it exhibits robust order parameters—ferroelectricity (**P**), antiferromagnetism (**L**), a weak ferromagnetic moment (**M**_**C**_) induced by canting of the antiferromagnetically aligned spins, and magnetoelectric coupling between these order parameters (**P**, **L**, **M**_**C**_) well above room temperature^3^. Because of the nature of its magnetoelectric coupling, BiFeO_3_ holds significant promise to trigger the development of low-power consumption memory and logic devices^4–6^. The strong spontaneous polarization in BiFeO_3_, however, results in a correspondingly large coercive voltage [described by the classic Landau double-well (Fig. 1a)], which requires adjustment to enable low-voltage applications^7,8^. Bulk BiFeO_3_ has a ferroelectric polarization of ~90 μC cm^−2^ pointing along the 〈111〉_pc_ (where pc refers to the pseudo-cubic notation) with a rhombohedrally distorted crystal structure (space group *R*3*c*)^9^. Unlike most conventional displacive ferroelectrics (e.g., BaTiO_3_ and PbTiO_3_) which have polarization induced by the hybridization between the empty transition metal *d* orbital and the filled oxygen 2*p*-orbital, the polarization in BiFeO_3_ primarily originates from the stereo-chemically active lone pair in the form of the *A*-site Bi^3+^ 6s^2^ electrons^10,11^. This has motivated – because of the intimate connection with the polarization – extensive studies of chemical substitution of the *A* site of BiFeO_3_ including using rare-earth elements such as La^3+^, Sm^3+^, and Dy^3+^ because of their similar ionic radius and isovalent chemistry to bismuth. A systematic change in ferroelectric ordering is indeed induced by rare-earth substitution for bismuth, including a reduction of the Curie temperature, formation of an antiferroelectric phase, etc^12–16^. On the other hand, isovalent chemical substitution of the *A* site is not expected to alter the antiferromagnetism in BiFeO_3_ and, in fact, only minimal effects on the Néel temperature are reported^17^. More importantly, however, is the impact of such rare-earth substitution for the evolution of the coupling mechanism between the magnetic and ferroelectric order. Despite having profound implications for material and device function, few studies have considered this. In turn, in the pursuit of the important technological question of how to enable low-voltage control of magnetism, foundational studies to unveil the correlation between **P**, **L**, **M**_**C**_, and their switching pathways in, for example, rare-earth lanthanum-substituted BiFeO_3_ (Bi_1−*x*_La_*x*_FeO_3_) thin films are a key step towards addressing the material requirements for low-power spintronics.

## Results

### Polarization reduction and rotation in Bi_1−*x*_La_*x*_FeO_3_

To study the evolution of the order parameters, **P**, **L**, and **M**_**C**_, under chemical pressure, we explore a model system consisting of (001)_pc_ oriented Bi_1−*x*_La_*x*_FeO_3_ (*x* = 0, 0.1, 0.15, 0.2, and 0.3) thin films grown on a conducting layer of SrRuO_3_ on insulating DyScO_3_ (110)_*O*_ single crystal substrates (where O refers to orthorhombic indices), all grown by pulsed-laser deposition (Methods). Rhombohedral BiFeO_3_ can be thought of as exhibiting a pseudocubic perovskite unit cell which has been distorted along the 〈111〉_pc_ body diagonal resulting in a spontaneous polarization along that axis. On the other hand, in Bi_1−*x*_La_*x*_FeO_3_, increasing amounts of lanthanum drives a suppression of the magnitude of and, possibly reorient, the polarization direction (Fig. 1b). As a consequence, chemical substitution could also provide a methodology to reduce the free-energy barrier for ferroelectric switching described by a classical Landau model (Fig. 1a) and experimentally demonstrated in polarization hysteresis loops (Fig. 1c). The polarization hysteresis loops, as a function of lanthanum content (Bi_1−*x*_La_*x*_FeO_3_ with *x* = 0, 0.1, 0.15, 0.20, and 0.3), reveal a systematic suppression of remanent polarization (from 65 µC cm^−2^ for *x* = 0 to 18 µC cm^−2^ for *x* = 0.2) and a corresponding reduction of the average coercive field (from 158 kV cm^−1^ for *x* = 0 to 106 kV cm^−1^ for *x* = 0.2).

Reciprocal space maps (RSMs) about the 203_pc_-diffraction condition of the films confirm the change in crystal structure (Supplementary Figs. 1, 2) and were used to extract the rhombohedral angles as well as the in-plane and out-of-plane lattice constants for this series of samples. We observe that the rhombohedral distortion decreases with lanthanum substitution while maintaining the imposed strain from the DyScO_3_ substrate (i.e., all films are coherently strained to the substrate such that the in-plane lattice constant of the film remains the same despite changing the chemistry). This indicates that a phase transition from rhombohedral to orthorhombic can be driven by such a chemical substitution^13^.

**Fig. 2:**
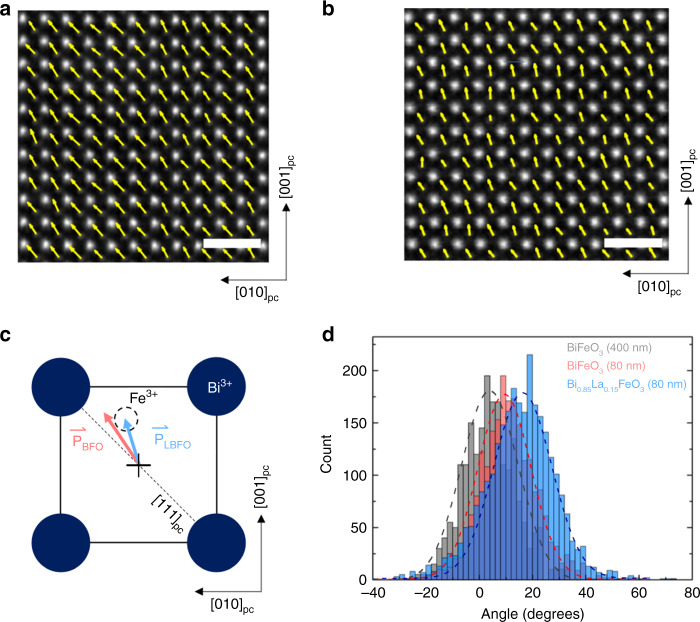
Atomic images, polarization mapping, and change of polarization in BiFeO_3_ and Bi_0.85_La_0.15_FeO_3_ thin films. **a**, **b** show the HAADF-STEM images of BiFeO_3_ and Bi_0.85_La_0.15_FeO_3_, respectively, with the polarization mapping of the Fe atoms overlaid. The scale bar is 1 nm. **c** Schematic of ferroelectric polarization in BiFeO_3_/Bi_0.85_La_0.15_FeO_3_ unit cell. The vectors in (**a**, **b**) were extracted from the displacement of Fe^3+^ position to the mass center of four Bi^3+^. **d** Histogram of polar distribution shows that the ferroelectric polarizations rotate 3.6°, 10.9° and 16.1° away from [111]_pc_ in 400-nm-thick BiFeO_3_ (gray), 80-nm-thick BiFeO_3_ (red) and 80-nm-thick Bi_0.85_La_0.15_FeO_3_ (blue), respectively.

While it is clear that lanthanum substitution can reduce the magnitude of the measured polarization and the coercive field, the specific nature of this reduction (i.e., does it arise from polarization rotation, reduction of polarization magnitude, or some combination therein) remains to be determined. To directly visualize the nature of the change in polarization at the microscopic scale, we carried out high-angle annular dark field scanning transmission electron microscopy (HAADF-STEM, “Methods” section) imaging on both the B_0.85_La_0.15_FeO_3_ and BiFeO_3_ thin films. From this, it is possible to extract quantitative local information about the direction of the polarization vector by measuring the displacement of the B-site cation (i.e., iron) to the mass center of the four cations at the unit-cell corners (i.e., bismuth and lanthanum) across each unit cell in the image (Fig. 2c). For BiFeO_3_ (Fig. 2a), the extracted map of polarization (which is a projection upon a (100)_pc_) reveals a strong tendency for the polarization to point along a diagonal direction – consistent with polarization pointing along a [111]_pc_ – but slightly tilted towards [001]_*pc*_ (as shown in Fig. 2d). (this is intuitively expected in the 0.4% compressively strained BiFeO_3_, which should drive a rotation of the polarization towards the out-of-plane direction ~10.9° from [111]_pc_ as shown in Fig. 2d)^18^ In contrast, for the Bi_0.85_La_0.15_FeO_3_ (Fig. 2b), the extracted map of polarization (which again is a projection on a (100)_pc_) reveals a clear rotation of the polarization away from a [111]_pc_ towards a [112]_pc_ by ~16.1° (the clockwise rotation is defined as positive direction), revealed by the histograms of measured polar vector directions across the entire area analyzed for both the BiFeO_3_ and Bi_0.85_La_0.15_FeO_3_ (Fig. 2d). We also measured the polarization rotation of the 400-nm-thick BFO sample where the polarization only deviates ~3.6° from [111]_pc_ (Fig. 2d). For reference, the angle between the [111]_pc_ and [112]_pc_ in this projection is 19.4°.

This, in conjunction with the experimentally measured remanent polarization in BiFeO_3_ and Bi_0.85_La_0.15_FeO_3_ projected along [001]_pc_ (Fig. 1c), provides us with an estimate of the ferroelectric polarization for such a structurally distorted and chemically substituted, Bi_0.85_La_0.15_FeO_3_ film (the details are captured in the Supplementary Information Fig. 1). We calculate the ferroelectric polarization in Bi_0.85_La_0.15_FeO_3_ to be ~44 µC cm^−2^, i.e., a significant reduction from the spontaneous polarization of 90 µC cm^−2^ in pure BiFeO_3_. Thus, we conclude that incorporation of 15% lanthanum into the parent BiFeO_3_ structure alters the nature of the polarization (i.e., both the magnitude and the direction of the polar vector), consistent with prior work^14–16,19^.

**Fig. 3:**
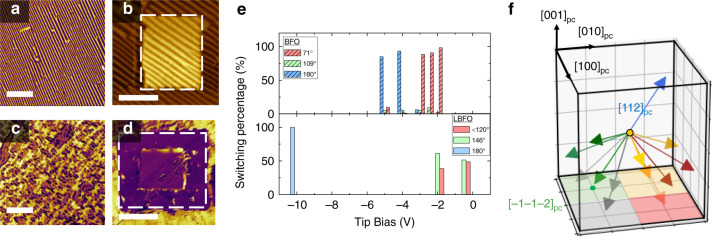
Ferroelectric switching in BiFeO_3_ and Bi_0.85_La_0.15_FeO_3_ revealed by PFM. **a**, **c** The as-grown IP-PFM images show different domain patterns in 20-nm-thick BiFeO_3_ and Bi_0.85_La_0.15_FeO_3_ thin films, respectively. **b** In-plane PFM images of 20-nm-thick BiFeO_3_ after PFM electric poling with −5 V (upward polarized). **d** In-plane PFM images of 20-nm-thick Bi_0.85_La_0.15_FeO_3_ after PFM electric poling with −10 V (upward polarized). The scale bar is 1 μm. **e** The summary of the polarization switching angles for 20-nm-thick BiFeO_3_ and Bi_0.85_La_0.15_FeO_3_ thin films. **f** Schematic of ferroelectric polarization switching in Bi_0.85_La_0.15_FeO_3_ thin film.

Armed with an understanding from X-ray diffraction and STEM analyses on how the material structure is evolving, we turned to piezoresponse force microscopy (PFM, “Methods” section) for an analysis of the evolution of the mesoscale domain structure, which similarly reveals marked differences. The as-grown domain structure of 20-nm-thick BiFeO_3_ features well-ordered stripe domain patterns (Fig. 3a), while that of 20-nm-thick Bi_0.85_La_0.15_FeO_3_ has no stripes, but exhibits a complex domain pattern (Fig. 3c). This is a consequence of weakened rhombohedral distortion (and a concomitant reduction in structural anisotropy), which is consistent with both the X-ray diffraction and STEM analyses above. In turn, one would expect that these crystal and domain structure differences will likely lead to corresponding differences in the mesoscale ferroelectric switching behavior – which can be probed via a combination of both lateral and vertical PFM (“Methods” section). For a 20-nm-thick BiFeO_3_ heterostructure (Fig. 3b), for example, application of a – 5 V DC voltage to the PFM tip reveals, as expected, classical 180° switching of polarization (i.e., reversed phase contrast in both the lateral and vertical PFM channels; Supplementary Information and Supplementary Figs. 3–6). For the same thickness Bi_0.85_La_0.15_FeO_3_ heterostructure (Fig. 3d), however, 180° switching of the polarization cannot be achieved unless a voltage very close to the breakdown voltage (~10 V, 5 MV cm^−1^) is applied. We capture the statistics of this difference in switching behavior in Fig. 3e, which shows histograms of the switching process as a function of voltage for both 20-nm-thick BiFeO_3_ and Bi_0.85_La_0.15_FeO_3_, from which a summary of the differences in switching behavior can be constructed (Fig. 3f). Unlike BiFeO_3_, Bi_0.85_La_0.15_FeO_3_ has the polarization vector pointing along ~〈112〉_pc_ resulting in a larger number of possible switching paths (e.g., for the 〈112〉_pc_, there are 12 possible switching directions (illustrated in Fig. 3f), of which only one corresponds to a full 180° switch; in comparison, there are only 4 possible switching directions for the 〈111〉_pc_ polarization direction in BiFeO_3_. The details are laid out in the Supplementary Information and Supplementary Fig. 4a. This high energy penalty of 180° switching in Bi_0.85_La_0.15_FeO_3_ could be associated with the energy consumption to accommodate ferroelastic deformation during multiple-step switching similar to that observed in BiFeO_3_^20^.

With this understanding of the nature of the ferroelectric polarization and its switching in Bi_0.85_La_0.15_FeO_3_, we now turn our attention to exploring the magnetic order, namely, **L** and **M**_**C**_. The parent phase BiFeO_3_ has a *G*-type antiferromagnetic structure, wherein the iron magnetic moments are ferromagnetically coupled within {111}_pc_ and antiferromagnetically aligned between adjacent {111}_pc_. Further, the anti-symmetric Dzyaloshinskii-Moriya exchange interaction (DMI), manifests from spin-orbit coupling and allows for the formation of a macroscopic magnetization (**M**_**C**_) caused by a canting of the antiferromagnetic sublattices^21^. The DMI has a simple energy expression:

1 $$E = - D \times \left( {L \times M_{\rm{C}}} \right),$$

where **D** is the DMI vector, **L** is the antiferromagnetic vector, and **M**_**C**_ is the canted magnetic moment vector^22^. In bulk BiFeO_3_, to minimize the free energy, the neighboring magnetic sublattices (**M**_**1**_, **M**_**2**_), which define the antiferromagnetic axis: **L** *=* **M**_**1**_ − **M**_**2**_, should be canted leading to a **M**_**C**_ that is perpendicular to **L** and rotates within the {111}_pc_ magnetic easy planes with a periodicity of ~62 nm due to the non-degeneracy of magnetic anisotropy^23^. We also note that this spin-cycloid structure is known to be suppressed in high-quality strained epitaxial thin films in which density functional calculations suggest that the **L** should lie along 〈110〉_pc_ or 〈112〉_pc_ with the {111}_pc_^22,24–26^. Regardless of the existence of the spin-cycloid structure, these three vectors, **D**, **L**, and **M**_**C**_ naturally form a right-handed multiferroic vector system (**D** aligns with 〈111〉_pc_, which is parallel to the ferroelectric polarization)^22^. This right-handed vector system is the crucial underpinning behind magnetoelectric coupling^20^. It has been shown that under large compressive strain (for example imposed by epitaxial strain from a SrTiO_3_ substrate [−1.4% compressive]), the single ion anisotropy component of the Hamiltonian can begin to dominate^27^; however, given the significantly smaller strain imposed by the DyScO_3_ substrate in our case, we expect the magnetic structure to be still controlled by the DMI in the crystal. With this as the background, an understanding of how the magnetic order parameters develop in Bi_0.85_La_0.15_FeO_3_ as a consequence of the significant rotation of the polarization vector away from the 〈111〉_pc_ direction is a critical and fundamentally important question. Specifically, we are interested in two questions: (1) do **P**, **L**, and **M**_**C**_ still form a right-handed system in Bi_0.85_La_0.15_FeO_3_? and (2) what happens to **P**, **L**, and **M**_**C**_ when the polarization is switched with an out-of-plane electric field?

### Probing magnetic order and magnetoelectric coupling in Bi_1−*x*_La_*x*_FeO_3_

**Fig. 4:**
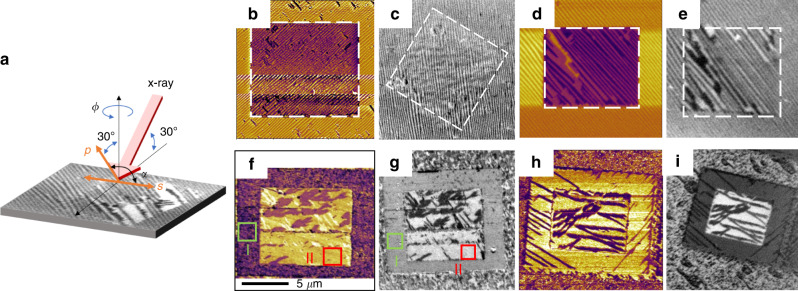
PFM and XMLD-PEEM images of BiFeO_3_ and Bi_0.85_La_0.15_FeO_3_ thin films. **a** Schematic of the XMLD-PEEM experimental geometries used to probe the angle dependence (***Φ***), linear dichroism. Linear polarizations: ***α*** = 0°; Linear polarization p: ***α*** = 90°. **b**, **c** In-plane-PFM and XMLD-PEEM images of 80-nm-thick BiFeO_3_. **d**, **e** In-plane-PFM and XMLD-PEEM images of 20-nm-thick BiFeO_3_. **f**, **g** In-plane-PFM and XMLD-PEEM images of 80-nm-thick Bi_0.85_La_0.15_FeO_3_. The green/red boxes represent the positive/negative polarized domain I/II. **h**, **i** In-plane-PFM and XMLD-PEEM images of 20-nm-thick Bi_0.85_La_0.15_FeO_3_.

To address these questions, we used X-ray magnetic linear dichroism-photoemission electron microscopy (XMLD-PEEM) combined with PFM to establish the correlation between the order parameters in Bi_1−*x*_La_*x*_FeO_3_ thin films. The geometry of the XMLD-PEEM experiment is shown with two angular dependences (***α*** for the linear polarization of the X-rays and ***Φ*** for the azimuthal in-plane direction of the sample; Fig. 4a). Here we used two types of samples, BiFeO_3_ and Bi_0.85_La_0.15_FeO_3_, with two different thicknesses, 20 and 80 nm (see sample preparation details in the Method section). Typical of high-quality BiFeO_3_ heterostructures, two-variant polarization, periodic stripe domains are observed for both 80 nm (Fig. 4b) and 20 nm (Fig. 4d) heterostructures^28^. We performed electrical switching of the ferroelectric domain structure (shown in the box) and, by careful control of the applied bias to the PFM tip (Methods and Supplementary Information), we are able to obtain stripy, two-variant domain structures after switching. Such a model system (both the 80- and 20-nm-thick BiFeO_3_ heterostructures) provides the reference frame to study the antiferromagnetic ordering **L**. XMLD-PEEM is a powerful tool for the investigation of magnetic ordering for many ferromagnetic and antiferromagnetic systems with high spatial resolution and elemental specificity^29–31^. This approach allows one to image the antiferromagnetic domains (Fig. 4c, e) with chemical specificity (here the images were taken at the iron *L*_*2*_ edge; Methods). This specific energy (peak) was chosen for imaging because both antiferromagnetic and ferroelectric ordering contribute strongly to the dichroism at the iron *L*_*3*_ edge while the dichroism at the *L*_*2*_ edge is dominated by magnetic order^25,32,33^. Consistent with previous studies, we observe a one-to-one correlation of ferroelectric and antiferromagnetic domains in both the 80- and 20-nm-thick BiFeO_3_ films due to the inherent coupling of polarization and magnetization in this material. By extracting the intensity of different ***α*** and ***Φ*** XMLD-PEEM images in different domains (see the analysis of **L** in Supplementary Information and Supplementary Figs. 7, 9), we find that the **L** in both the 80- and 20-nm-thick BiFeO_3_ heterostructures is approximately parallel to a 〈112〉_pc_ before and after electrical switching.

Similar analysis was completed on the Bi_0.85_La_0.15_FeO_3_ heterostructures, which also show electrically switched antiferromagnetic domains with one-to-one correlation to the ferroelectric domains (Fig. 4f–i). Fitting of the XMLD intensity versus ***α*** and ***Φ*** in the antiferromagnetic domains for Bi_0.85_La_0.15_FeO_3_ (Supplementary Figs. 8–10), however, show that the **L** in domain I is along ~ [641]_pc_ and the **L** in domain II is along ~ [10–2]_pc_, which means the **L** rotates by ~105°, consistent with the **P** switching (Fig. 3f). This also points to the fact that the easy magnetic plane for Bi_0.85_La_0.15_FeO_3_ is no longer in the {111}_pc_ but most likely in the {121}_pc_ (Supplementary Fig. 11). We attribute this change in the magnetic easy plane to the consequence of the rotation of the ferroelectric polarization in the Bi_0.85_La_0.15_FeO_3_ (Fig. 1). That is, as a consequence of the polarization in Bi_0.85_La_0.15_FeO_3_ pointing (approximately) along the [112]_pc_, **L** and **M**_**C**_ are expected to follow the motion of the polarization accordingly (i.e., polarization is switched from, for example, [1-12]_pc_ to [21-1]_pc_).

Based on the ferroelectric data in Fig. 3 and the antiferromagnetic data in Fig. 4, one can calculate the canted moment direction, **M**_**C**_, using eq. (1). Our calculations indicate that the canted moment direction switched from **M**_**CI**_ ~[−7 13 10]_pc_ to **M**_**CII**_ ~[251]_pc_ with the applied electric field in the Bi_0.85_La_0.15_FeO_3_ samples, while in BiFeO_3_ samples, the canted moment stays in the plane (110)_pc_. This can be validated by exploring the coupling of the **M**_**C**_ to an external magnet. For example, an in-plane magnetized Co_0.9_Fe_0.1_ layer shows a progressively lower exchange coupling field, as illustrated in Supplementary Fig. 12, supporting the notion that the canted moment is tilting away from the in-plane direction towards the out-of-plane, as lanthanum is added. In contrast, a ferromagnetic multilayer with perpendicular magnetic anisotropy, such as Co/Pt multilayer, shows a stronger out-of-plane magnetic anisotropy on the Bi_0.85_La_0.15_FeO_3_ compared to the BiFeO_3_, Supplementary Fig. 13. Both these pieces of data provide substantiation that the out-of-plane magnetic exchange coupling is enhanced in the Bi_0.85_La_0.15_FeO_3_ sample; we attribute this enhancement to tilting of **M**_**C**_ as a consequence of lanthanum substitution in BiFeO_3_.

### Magnetoresistance measurements and micromagnetic simulations

**Fig. 5:**
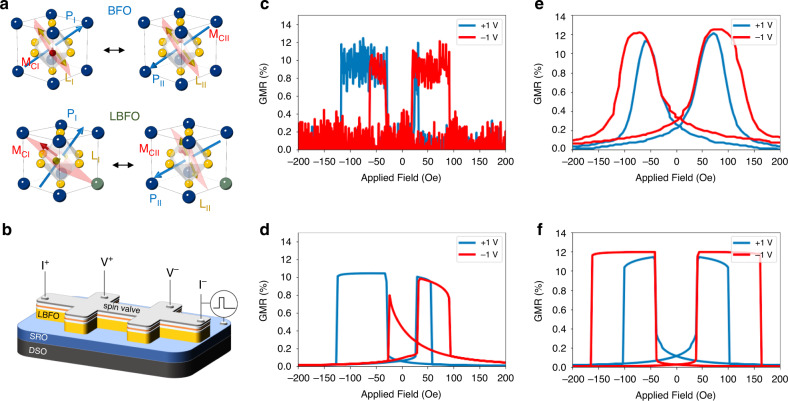
Magnetic anisotropy switching by electric field via the heterostructure of spin-valve/BiFeO_3_ (Bi_0.85_La_0.15_FeO_3_). **a** Schematics for the different electrically polarized states of **P**, **L**, and **M**_**C**_ in BiFeO_3_ and Bi_0.85_La_0.15_FeO_3_. **b** Schematic for magnetoresistance measurements on spin-valve/BiFeO_3_ (Bi_0.85_La_0.15_FeO_3_) heterostructure. **c** R(H) of spin-valve/BiFeO_3_ on the different electrically polarized states. **d** micromagnetic simulations on R(H) of spin-valve/BiFeO_3_ with different polarization states. **e** R(H) of spin-valve/ Bi_0.85_La_0.15_FeO_3_ on the different electrically polarized states. **f** Micromagnetic simulations on R(H) of spin-valve/ Bi_0.85_La_0.15_FeO_3_ with different polarization states.

Having established the changes in **P**, **L**, and **M**_**C**_ as a function of lanthanum substitution as well as with electric field applied, captured schematically in Fig. 5a, we now proceed to ask the question: how do they impact the magnetotransport behavior of a spin-valve that is in magnetic contact with the Bi_0.85_La_0.15_FeO_3_ surface. We deposited a spin-valve composed of Pt (2 nm)/Co_0.9_Fe_0.1_ (2.5 nm)/Cu (5 nm)/Co_0.9_Fe_0.1_ (2.5 nm) on the Bi_0.85_La_0.15_FeO_3_ surface and fabricated Hall-bar structures (Fig. 5b) to study the electrical-field dependence of magnetoresistance [R(H)] which is measured as the applied magnetic field, *H*, is swept from the positive value *H*_*max*_ to the negative value −*H*_*max*_ and back to zero field (sample preparation and device fabrication details are presented in the Methods section). We use the conventional form of magnetoresistance, defined as GMR (%) = [*R*(*H*) − *R*(*H*_max_)] × 100% / *R*(*H*_max_). To draw the distinction, we compare a pure BiFeO_3_ layer to the Bi_0.85_La_0.15_FeO_3_ layer of the same thickness, Fig. 5c–f. We support the experimental data through micromagnetic simulations (details presented in Supplementary Information and Method section).

A typical GMR hysteresis loop as a function of electric bias (see the transport measurement details in Method Section) for the spin-valve on the pure BiFeO_3_ is shown in Fig. 5c, with the corresponding micromagnetic simulation in Fig. 5d. Similarly, the GMR hysteresis loop for the spin-valve on Bi_0.85_La_0.15_FeO_3_ is shown in Fig. 5e, with the simulated data in Fig. 5f. For BiFeO_3_, we observe the modulation of an exchange bias by the applied electric field, which is consistent with our previous study^34^; on the other hand, for the Bi_0.85_La_0.15_FeO_3_ case, we observe the modulation of exchange coupling, leading to a reduction of the GMR switching field at positive electric field (additional details presented in Supplementary Fig. 14).

We can now put the data presented in Figs. 3–5 together to present the following observations. The insertion of lanthanum into BiFeO_3_ leads to a rotation of both **P** and **L** as well as their significantly different switching behaviors with an electric field. In the case of Bi_0.85_La_0.15_FeO_3_, the rotation of **M**_**C**_ by ~105° amounts to an effective change in the magnetic anisotropy from essentially in-plane to essentially out-of-plane. This rotation of the magnetic anisotropy in Bi_0.85_La_0.15_FeO_3_ explains the E-field modulation of the exchange coupling between the Co_0.9_Fe_0.1_ and the Bi_0.85_La_0.15_FeO_3_ layer that can be described by eq. (2). From Eq. (2), one can approximately derive the contributions to the energy of Co_0.9_Fe_0.1_ resulting from its interaction with Bi_0.85_La_0.15_FeO_3_:

2 $$F_{FM - AFM} = \mu _0H_{eb}M_c\left( {\hat m_c \cdot \hat m} \right) + K_{ec}\left( {\hat l \cdot \hat m} \right)^2$$

Here the lower-case symbols with a hat designate normalized vectors. Even though the values of exchange bias **H**_eb_ and exchange coupling *K*_ec_ can be expressed via the exchange energy, their phenomenological values are more reliable, since they account for non-idealities of the interface. From the expression of exchange coupling energy, we can foresee that once we can control the anisotropy in the ferromagnet through the magnetoelectric coupling in Bi_0.85_La_0.15_FeO_3_, we will be able to switch its magnetization. This switch can be detected by measuring GMR in the spin-valve.

The micromagnetic simulations of the GMR stack (all the simulation details can be found in the Method and Supplementary Information) presented in Fig. 5d and f show a close resemblance to the experimental data and provide theoretical credence to these experimental observations above. Compared to the parent phase BiFeO_3_, Bi_0.85_La_0.15_FeO_3_ shows a very different magnetoelectric switching where the magnetic anisotropy (mainly the magnetic easy plane) can be controlled by electric field while BiFeO_3_ has the same magnetic easy plane before and after the electric field is applied^25,32,34^.

## Discussion

In summary, we have discovered that the polarization vector in the BiFeO_3_ system is systematically varied in both its magnitude and direction with respect to the crystal lattice, as the Bi^+3^ is replaced by La^+3^. The rotation of the polar vector away from the 〈111〉_pc_ direction and towards the 〈112〉_pc_ direction appears to be critical in terms of understanding both the anisotropy energy of the polar phase as well as the response of the system to out of plane electric fields. In addition, we demonstrate the ability for electrical control the anisotropy of magnetoelectric coupling through a spin-valve/Bi_0.85_La_0.15_FeO_3_ device. Our results fulfill the understanding of magnetoelectric coupling in chemically-modified BiFeO_3_ thin films, and can trigger the new application of multiferroics.

## Methods

### Film growth

The oxide heterostructures Bi_0.85_La_0.15_FeO_3_/SrRuO_3_ or BiFeO_3_/SrRuO_3_ are grown on single-crystalline (110)_O_ DyScO_3_ by pulse laser deposition at 690–710 °C with focused laser fluence ~1.2 J cm^−2^ under 100–160 mTorr oxygen pressure and cooled down to room temperature at 500 Torr oxygen pressure. After the cooling process, the oxide heterostructures were immediately transferred to high vacuum magnetron sputtering chamber with a base pressure ~1 × 10^−7^ Torr. The spin-valve structures we used in this article, are fabricated with Pt (2.5 nm)/Co_0.9_Fe_0.1_ (2.5 nm)/Cu (3–5 nm)/Co_0.9_Fe_0.1_(2.5 nm), deposited by DC magnetron sputtering with argon pressure from ranging from 2 × 10^−3^ to 7 × 10^−3^ Torr under a static magnetic field of 200 Oe along the crystallographic [001]_O_ to establish the magnetic easy axis. A 2 nm Pt layer is deposited on top of the spin-valve as a capping layer to protect the top Co_0.9_Fe_0.1_ layer from oxidation, whereas the bottom SrRuO_3_ layer serves as a bottom electrode for ferroelectric switching. The chemical composition analysis can be found in Supplementary Fig. 15.

### Scanning transmission electron microcopy and polarization mapping

TEM samples were prepared by mechanical polishing with an Allied Multiprep followed by ion milling in a Gatan PIPS2. Samples were prepared so that the projected plane was (1,0,0)c. HAADF-STEM images were collected on a JEOL JEM-ARM300CF operating at 300 kV.

In quantifying the polarization, we use the pseudocubic unit cell projected along the beam direction. The atomic polarization vector was calculated by measuring the displacement of B/A site atoms from the center of the four surrounding A/B site atoms in HAADF-STEM images. Each atom in the image is located accurately using gaussian fitting and the center of each unit cell is defined as the average position of the four atoms at the corners of the unit cell. Polarization mapping was performed on several images from different regions for the BiFeO_3_ and Bi_0.85_La_0.15_FeO_3_ samples. In bulk BiFeO_3_, the polarization in the (100)_pc_ projection plane should appear along the unit cell diagonal, [001]_pc_. To determine the deviation of the average polarization angle from the diagonal, we combined the data from several images together to generate one distribution for each sample. A Gaussian distribution was then fit to quantify the mean and standard deviation of the polarization angle in each sample.

### Fabrication of spin-valve devices and magnetoelectric coupling measurements

Conventional photolithography and Ar-ion milling were employed to patterned the Co_0.9_Fe_0.1_(2.5 nm)/Cu(5 nm)/Co_0.9_Fe_0.1_(2.5 nm) spin-valves devices of 15 × 5 µm^2^. Subsequently, a 200 nm thick insulating amorphous LaAlO_3_ film was selectively deposited on Bi_0.85_La_0.15_FeO_3_ surface by PLD to isolate the connecting lines and contact pads from Bi_0.85_La_0.15_FeO_3_. DC magnetron sputtering was used to deposit 230 nm thick Au film for electric contacts. The GMR responses of spin-valve devices were measured in current-in-plane configuration at constant current with varying magnetic field. The magnetoelectric coupling measurements were conducted with the application of electric pulse (10–100 µs) across Bi_0.85_La_0.15_FeO_3_ films.

### Photoemission electron microscopy (PEEM)

X-ray imaging with variable circular and linear polarization at the Co and Fe *L* edges was performed at the PEEM3 end station of BL11.0.1 at the Advanced Light Source, Lawrence Berkeley National Lab. The sample was held at an angle of 60° with respect to the surface normal, and was mounted on an azimuthal rotation stage. The in-plane azimuthal angle of zero degrees is with x-rays incident along the in-plane [−100]_pc_ direction (***Φ*** = 0°). The sample was held at a voltage of −18 kV to accelerate any photo-emitted and secondary electrons, proportional to the local x-ray absorption coefficient, through a series of electrostatic lenses and incident onto a CCD detector with a phosphor-coated fiber plate serving as an amplifier. To probe antiferromagnetic orientation projections along the x-ray linear polarization axis, linear dichroism images at the Fe *L*_2_ A and B edges of 720.6 and 722 eV were taken at 5 linear polarization axis angles between s- and p-polarization (s: ***α*** = 0°; p: ***α*** = 90°). For each polarization value, a pre-edge image at 718 eV was taken to normalize the on-edge images. A series of 10 images with 2 s acquisition time each were taken at each energy and polarization angle, and the entire polarization angle-energy sequence was repeated four times to improve statistics.

### Piezoresponse force microscopy (PFM)

PFM is performed with an Atomic Force Microscope (Asylum Research Cypher, Santa Barbara, Ca), conductive AFM probe (Nanoandmore, DT-NCHR, Watsonville, Ca) with DART mode. To determine domain orientation directions the normal (z) and lateral (y axis only) piezo-response vectors are measured simultaneously by superimposing two small-signal AC biases with distinct frequencies corresponding to normal and lateral contact resonances of the AFM cantilever (approximately 300 and 600 kHz, respectively). The essentially binary phase signals are then vectorially combined to identify the four domain orientations present in the specimen.

### Micromagnetic simulations

Micromagnetic simulations have been performed using the National Institute of Standards and Technology simulator, OOMMF. The inputs scripts for simulations are available in the Supplementary information. The simulation method follows closely to ref. ^34^. Magnetization is calculated by minimizing the overall energy of the system at every value of the external magnetic field.

In contrast to BiFeO_3_ which exhibits periodic stripe domains, Bi_0.85_La_0.15_FeO_3_ has wide domains of single polarization. Thus, the antiferromagnetic order, **L** and canted magnetization, **M**_**C**_, are assumed to be constant over the area of the device. The long axis of the measured spin-valve is along the [100]_pc_ crystal axis. To make simulations run in an acceptable amount of computing time, the geometry of the Co_0.9_Fe_0.1_ layer was taken to be smaller than in experiments: 2000 × 200 × 1 nm^3^ with the in-plane grid of 20 nm. This is justified since the contribution of the demagnetization (i.e. shape anisotropy) is small for this size range. To compensate for the ratio of the size to the exchange length in the ferromagnet, a lower value of the exchange stiffness was taken, $A_x = 10pJ/m$. Other parameters: $M_s = 1MA/m$ - magnetization of Co_0.9_Fe_0.1_, $K_{ec} = 5000J/m^3$ - in-plane anisotropy in the bottom layer due to the exchange coupling, exchange bias $H_{eb} = 0oe$. The normalized vectors mentioned above and used in simulations were at positive applied voltage *P*_I_ = [0.4082 −0.4082 0.8165]; *L*_I_ = [0.8242 0.5494 −0.1374]; *M*_CI_ = [−0.3925 0.7290 0.5608] and at negative applied voltage *P*_II_ = [−0.8165 0.4082 −0.4082]; *L*_II_ = [0.4472 0 −0.8944]; *M*_CII_ = [−0.3651 −0.9129 −0.1826]. One can see that the projection of the canted magnetization on the shorter in-plane axis of the spin-valve is smaller than the long axis of the spin-valve. This explains why the manifestation of the exchange bias in the asymmetry of the hysteresis loop is smaller here than in ref. ^34^.

## Supplementary information

Supplementary Information

## Data Availability

The data that support the findings of this study are available from the corresponding author upon reasonable request.
