# Supplementary material for: Manipulating magnetoelectric energy landscape in multiferroics
Source: Nat Commun. 2020 Jun 5;11:2836. doi: 10.1038/s41467-020-16727-2 (PMC7275047; doi:10.1038/s41467-020-16727-2)
Supplement: Supplementary file 1 — Supplementary Information [file 41467_2020_16727_MOESM1_ESM.pdf]

SUPPLEMENTARY INFORMATION

Manipulating Magnetoelectric Energy Landscape in Multiferroics

Y. L. Huang et al.

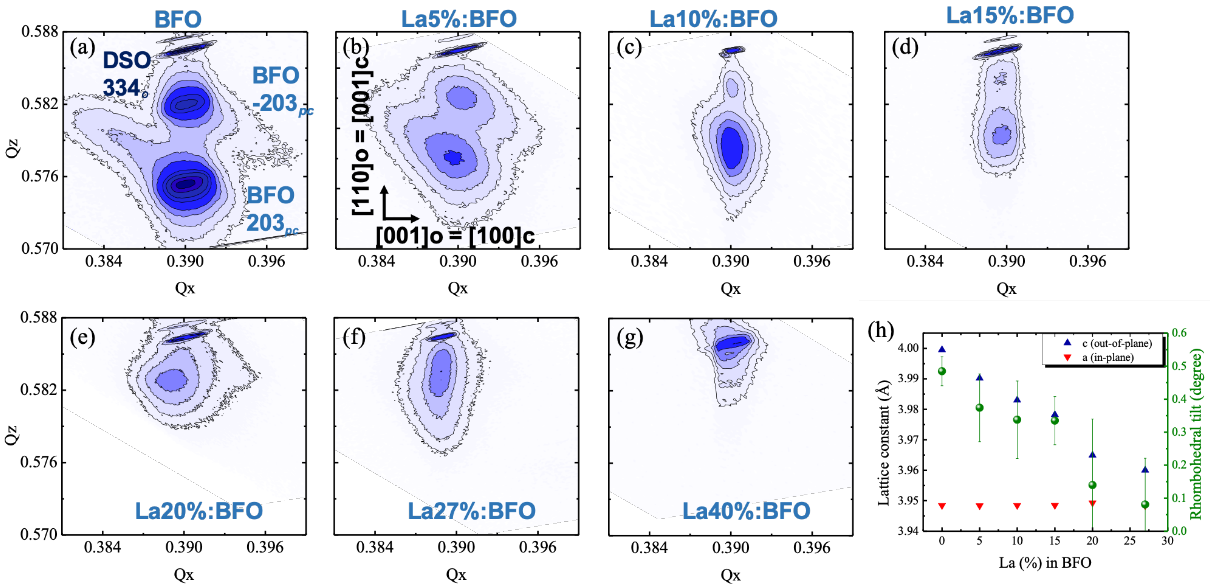

Supplementary Figure 1. RSMs of $Bi_{1-x}La_xFeO_3$ thin films in the $(203)_{pc}$ diffraction condition. (a) $BiFeO_3$, x = (b) 0.05, (c) 0.1, (d) 0.15, (e) 0.2, (f) 0.27, (g) 0.4, (h) The summary of the in-plane (shown in red), out-of-plane lattice parameters (shown in blue), and rhombohedral angles (shown in green).

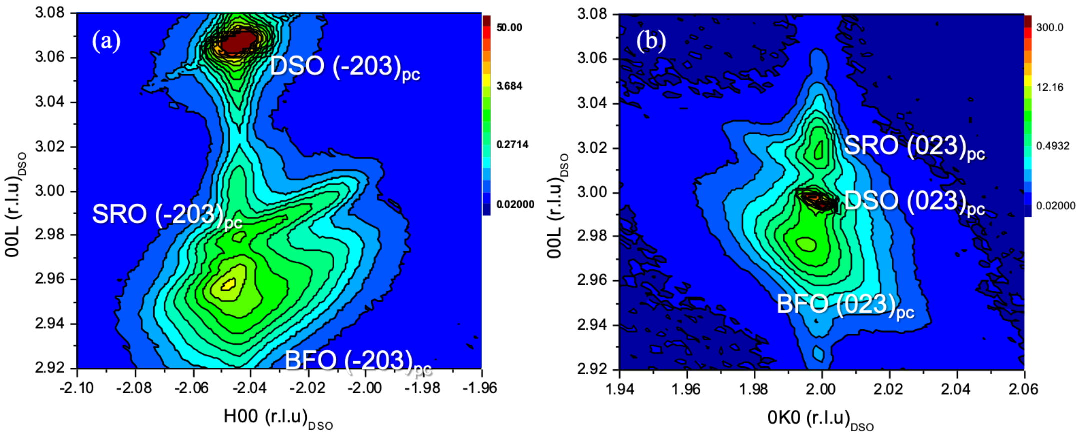

Supplementary Figure 2. RSM of $Bi_{0.8}La_{0.2}FeO_3$ thin films in the $(-203)_{pc}$ and $(023)_{pc}$ diffraction condition. With these high resolution RSMs, we can determine that the $Bi_{0.8}La_{0.2}FeO_3$ is not rhombohedral but orthorhombic.

Determination of P in $Bi_{0.85}La_{0.15}FeO_3$

The measured remanent polarization in $Bi_{0.85}La_{0.15}FeO_3$ is 35 μC/cm^2 along the out-of-plane direction, $[001]_{pc}$. With the measured polarization angle (tilting 16.9° from $[111]_{pc}$, the angle between $[001]_{pc}$ and $[111]_{pc}$ is 54.7°) from the STEM images (Figure 2), we can estimate the ferroelectric polarization in $Bi_{0.85}La_{0.15}FeO_3$:

$$P \times cos(36.8°) = 35\ \mu C/cm^2; \quad (1)$$

$$P = \frac{35}{cos(36.8°)} \mu \frac{C}{cm^2} = 44\ \mu C/cm^2; \quad (2)$$

Thus, the calculated **P** in the $Bi_{0.85}La_{0.15}FeO_3$ thin film is ~ 44 μC/cm^2.
To resolve **P** within an individual domain of $Bi_{0.85}La_{0.15}FeO_3$, we have to make certain assumptions:

1. The $Bi_{0.85}La_{0.15}FeO_3$ has **P** pointing along (and only along) $[112]_{pc}$.
2. The PFM signal can be demodulated into amplitude and phase from the ac-modulated signal at the off-contact-resonance frequency (typically 600 kHz). The amplitude is proportional to the **P** projected on either in-plane or out-of-plane direction. For the in-plane PFM signal, the amplitude is proportional to the projection onto the cantilever.

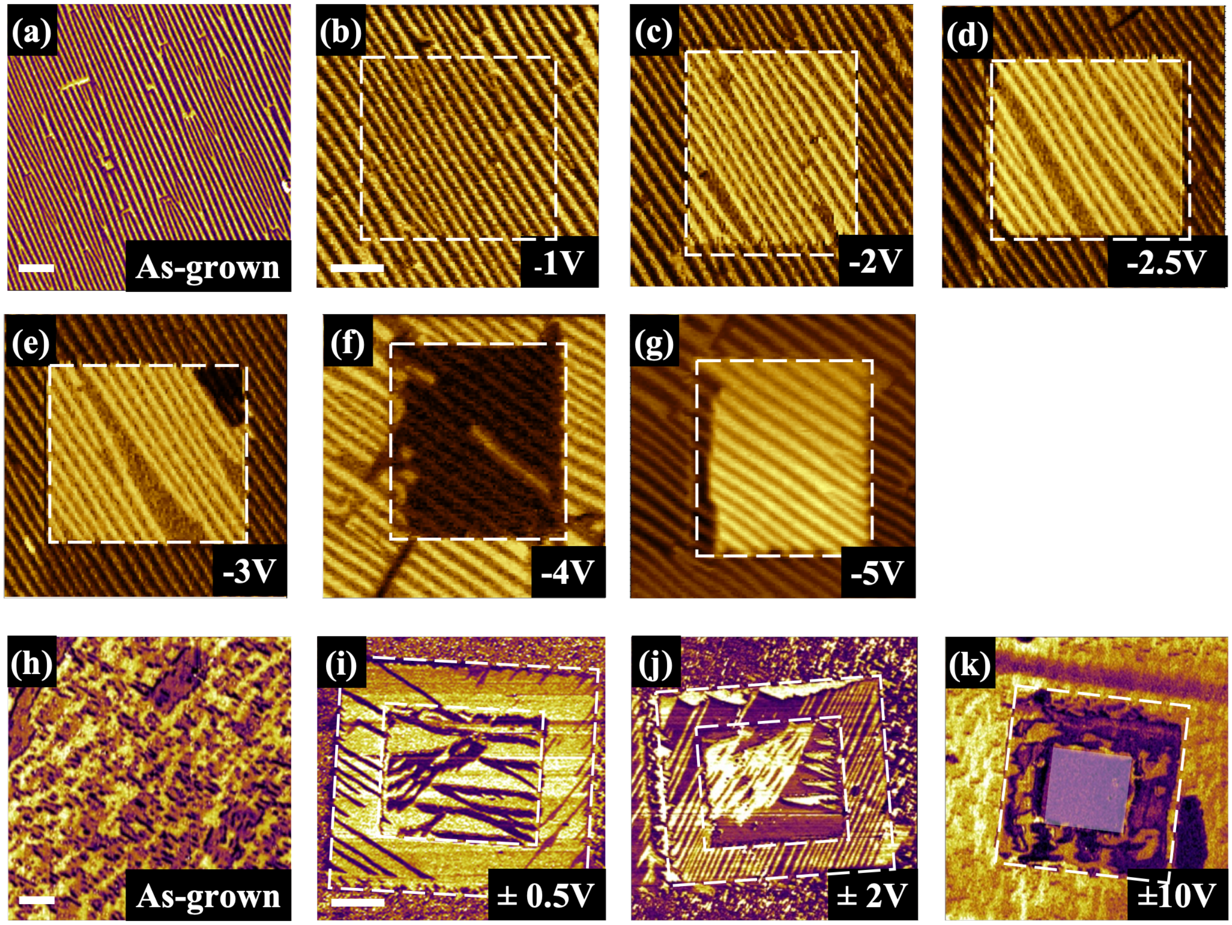

Supplementary Figure 3-1. Ferroelectric switching in $BiFeO_3$ and $Bi_{0.85}La_{0.15}FeO_3$ revealed by in-plane PFM. (a) and (h) The as-grown PFM images show different domain patterns for 20-nm-thick $BiFeO_3$ and 20-nm-thick $Bi_{0.85}La_{0.15}FeO_3$, respectively. In-plane PFM images of 20-nm-thick $BiFeO_3$ after PFM electric poling with (b) -1 V, (c) -2 V, (d) -2.5 V, (e) -3 V, (f) -4 V, and (g) -5 V. In-plane PFM images of 20-nm-thick $Bi_{0.85}La_{0.15}FeO_3$ after PFM electric poling

with (i) the outer box +0.5 V; the inner box: -0.5 V, (j) the outer box +2 V; the inner box: -2 V, (k) the outer box +10 V; the inner box: -10 V. The inner box with -10V shows dielectric break down with low piezoelectric response. The high voltage requirement for the 180° switching in BLFO sample can be related to the ferroelastic switching; for example switching from $[112]_{pc}$ to $[-1-1-2]_{pc}$ in two steps; ($[112]_{pc}$ to $[2-1-1]_{pc}$ and then to $[-1-1-2]_{pc}$). On the other hand, the 180° switching in pure BFO is a combination of 109° and 71° switching, in which the crystal still possesses the same deformation axis along $<111>_{pc}$; for example from $[111]_{pc}$ to $[11-1]_{pc}$ and then to $[-1-1-1]_{pc}$.

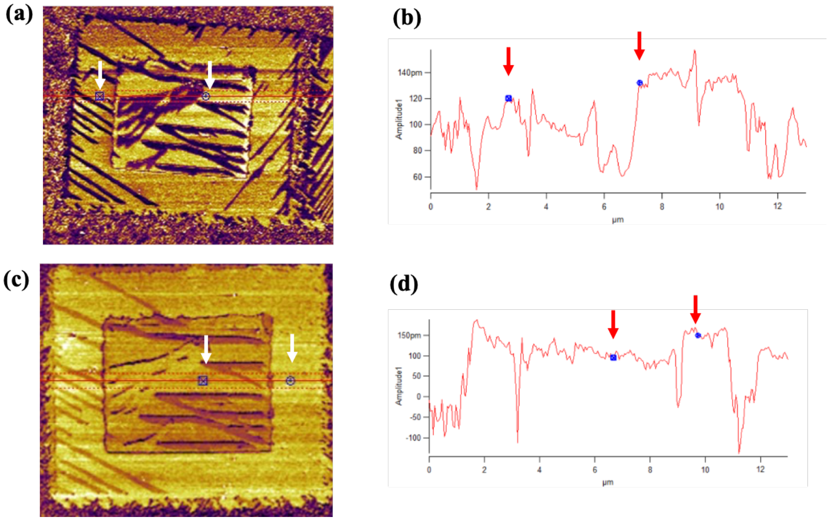

Supplementary Figure 3-2. Ferroelectric switching in $Bi_{0.85}La_{0.15}FeO_3$ revealed by out-of-plane and in-plane PFM. (a) The in-plane amplitude image of $Bi_{0.85}La_{0.15}FeO_3$ (the same area as Supplementary Figure 3-1 f). (b) The extracted signal line profile in (a). (c) The out-of-plane amplitude image of $Bi_{0.85}La_{0.15}FeO_3$ (the same area as Supplementary Figure 3f). (d) The extracted signal line profile in (c). For the case of switching from $[112]_{pc}$ to $[121]_{pc}$, the $[112]_{pc}$ type domain will possess a stronger out-of-plane signal and weaker in-plane amplitude signal and the other way around for the $[121]_{pc}$ type domain. If the switching belongs to the $[112]_{pc}$ to $[-1-1-2]_{pc}$ type, then both in-plane and out-of-plane amplitude channel should show the same signal magnitude.

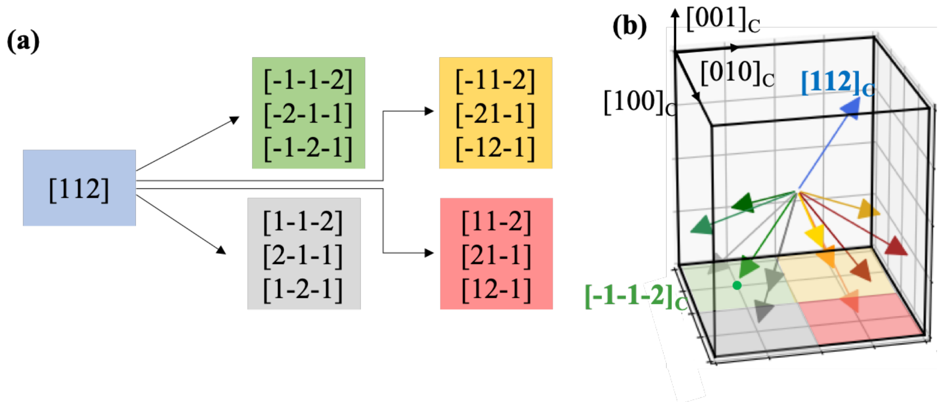

Supplementary Figure 4. (a) Ferroelectric switching pathways and (b) the corresponding schematic.

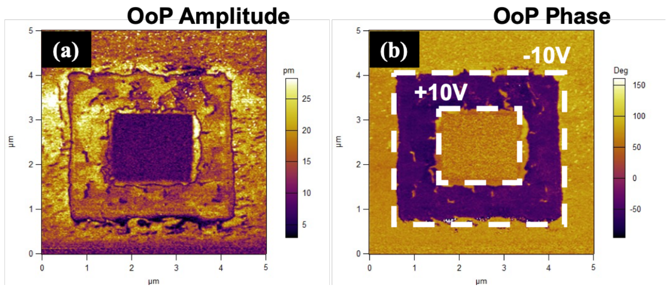

Supplementary Figure 5. Out-of-plane PFM images of (a) amplitude, and (b) phase channels. The inner box (written at +10 V) shows the features with minimal piezo-response attributable to dielectric breakdown. Here, we just focus on the comparison of amplitude inside and outside of the box (written at -10 V). For the 180° switching, the out-of-plane amplitude should have the same value and the phase should show a 180° difference. On the other hand, the in-plane amplitude channel should also show the same amplitude with a 180° phase difference.

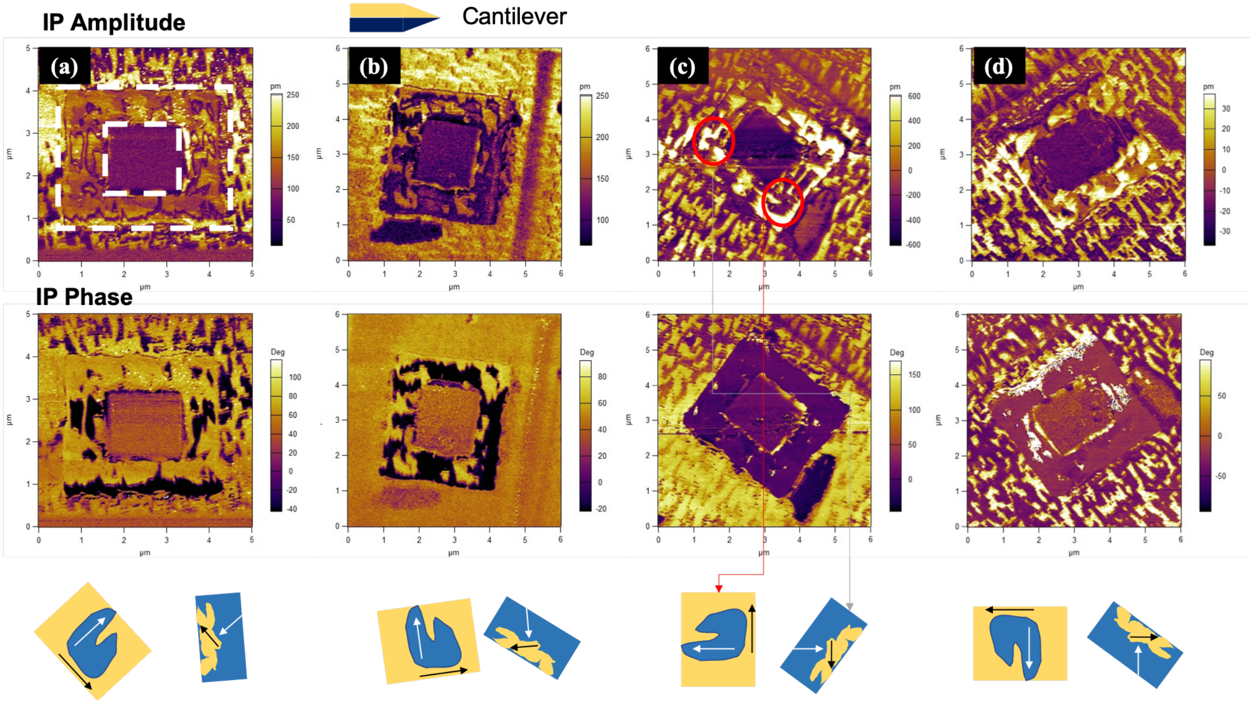

Supplementary Figure 6. In-plane PFM images of 20-nm-thick $Bi_{0.85}La_{0.15}FeO_3$ with different sample-cantilever configuration. (a) [100]$_{pc}$// (b) [100]$_{pc}$ ⊥ (c) 45° and (d) 135° to the cantilever, respectively. Upper panels show the amplitude images, middle panels show the in-plane phase images, and the bottom panels show the polarization direction in the given domains corresponding to the red circles in (c).

Determination of L in $BiFeO_3$ and $Bi_{0.85}La_{0.15}FeO_3$

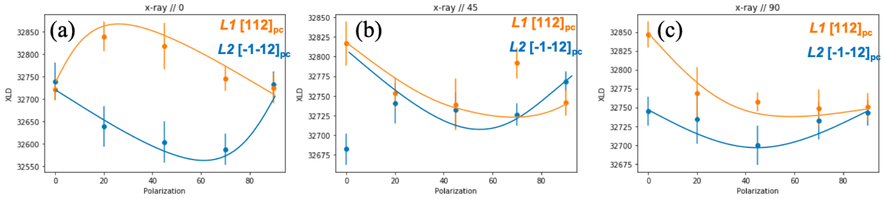

Supplementary Figure 7. Angle (α,ϕ) dependence of XMLD intensity of a 80-nm-thick $BiFeO_3$ thin film. (a) ϕ = 0°, (b) ϕ = 45°, (c) ϕ = 90°. The orange (blue) lines represent the guide to the eye as [112]$_{pc}$ ([-1-12]$_{pc}$) simulated XMLD signals using the equation:

$$I = (3\,cos^2\,\theta_M - 1) \times M^2$$

where θ is the angle between antiferromagnetic axis and the linear polarized x-ray, and M is the magnetization[1].

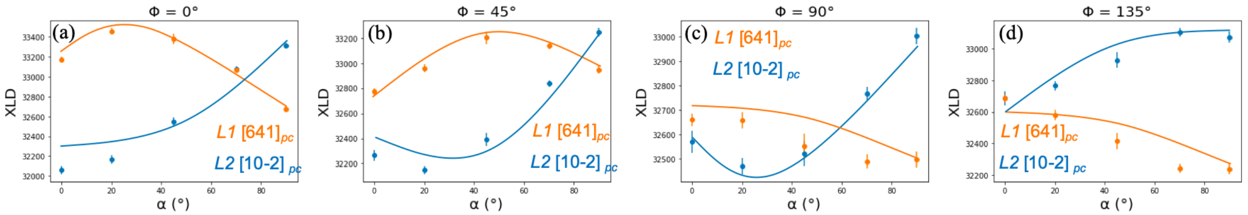

Supplementary Figure 8. Angle (α,ϕ) dependence of XMLD intensity of 20-nm-thick $Bi_{0.85}La_{0.15}FeO_3$ thin films. (a) ϕ = 0°, (b) ϕ = 45°, (c) ϕ = 90°, (d) ϕ = 135°. The antiferromagnetic axis in domain I and domain II: L_1 // [641]$_{pc}$ and L_2 // [10-2]$_{pc}$. We note that 80-nm-thick $Bi_{0.85}La_{0.15}FeO_3$ thin films show the same behavior.

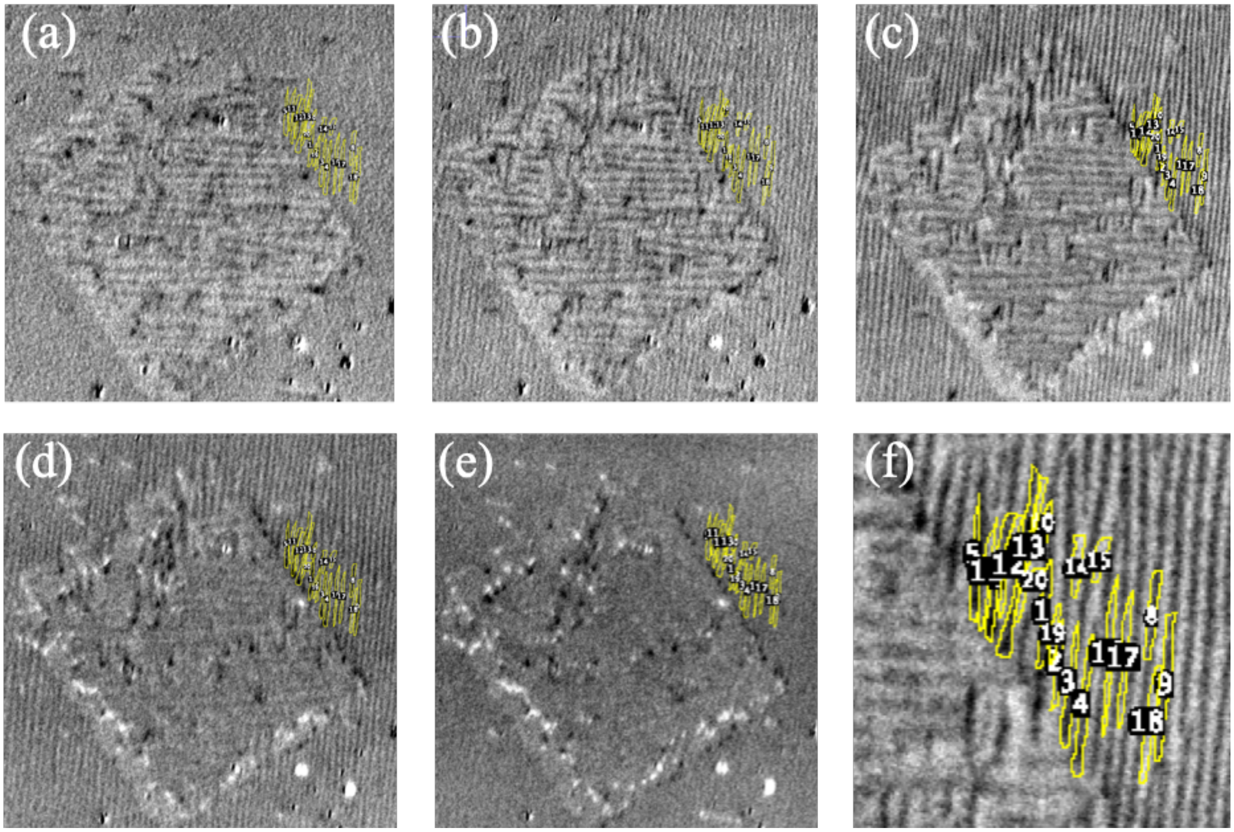

Supplementary Figure 9-1. Typical example of angle (α,ϕ = 90°) dependence of XMLD images of a 80-nm-thick $BiFeO_3$ sample. (a) α = 0°, (b) α = 20°, (c) α = 45°, (d) α = 70°, (e) α = 90°. The two-variant stripy domains are marked by the yellow contour to track the XMLD intensity in each image and plotted in Supplementary Figure 7. These XMLD images were taken from the difference between Fe L_2 resonance A and B peaks at room temperature[1]. (f) A zoom-in image of (c) as an example to demonstrate how we track the XMLD intensity in a particular domain pattern.

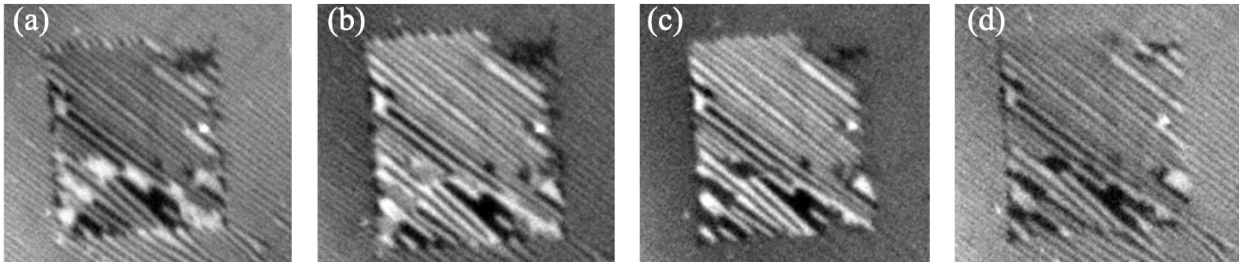

Supplementary Figure 9-2. Typical example of angle (α,ϕ = 90°) dependence of XMLD images of a 20-nm-thick $BiFeO_3$ sample. (a) α = 20°, (b) α = 45°, (c) α = 70°, (d) α = 90°.

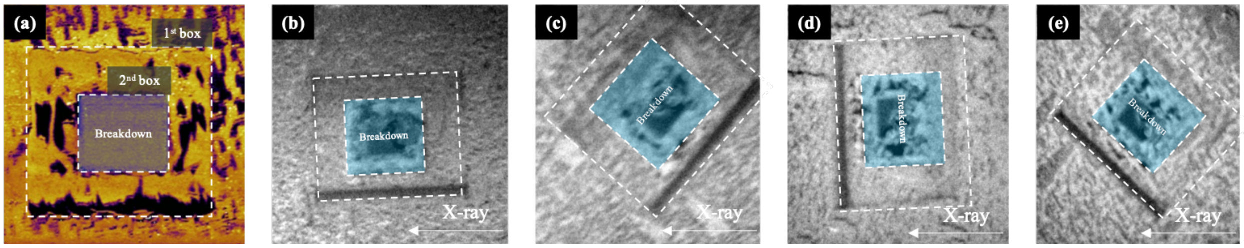

Supplementary Figure 10. Angle dependent XMLD-PEEM images of ±10V switched domains in a 20-nm-thick $Bi_{0.85}La_{0.15}FeO_3$ sample. (a) In-plane PFM phase image. The 2nd switched box indicates dielectric break down. The angle-dependence of incident x-ray images: $[100]_{pc}$ (b)// (c)45° (d) ⊥ and (e) 135° to the x-ray with *s*-polarization (shown in the Figure 3a). By comparing the XMLD intensity inside and outside the 1st box, we can discern that the antiferromagnetic axis **L** does not change by application of an electric field. This is because a 180° switching of **L** does not change the in-plane or the out-of-plane projection of the antiferromagnetism and thus cannot be detected by the XMLD-PEEM technique.

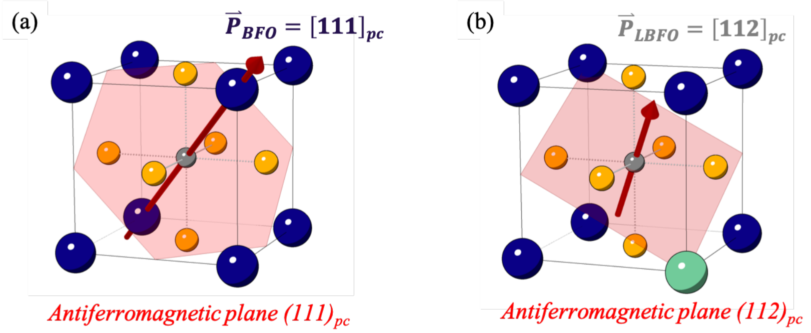

Supplementary Figure 11. Schematics for the correlation of magnetic easy plane and ferroelectric polarization in (a) $BiFeO_3$ and (b) $Bi_{0.85}La_{0.15}FeO_3$.

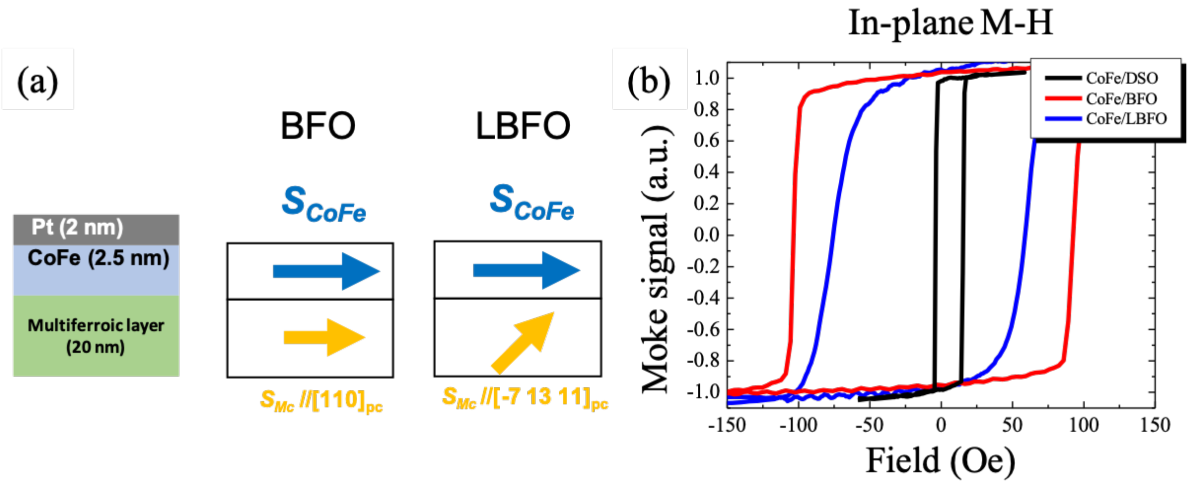

Supplementary Figure 12. Magnetic hysteresis loops of the $Co_{0.9}Fe_{0.1}$/$BiFeO_3$, $Co_{0.9}Fe_{0.1}$/ $Bi_{0.85}La_{0.15}FeO_3$, and $Co_{0.9}Fe_{0.1}$/$DyScO_3$ heterostructures measured along the in-plane direction. (a) Schematic of ferromagnet/multiferroic heterostructure. Also shown is a schematic description of the exchange coupling between the canted moment, $\mathbf{M_C}$ in the $BiFeO_3$ (or $Bi_{0.85}La_{0.15}FeO_3$) layer and the moment in the ferromagnet, $Co_{0.9}Fe_{0.1}$. (b) In-plane *M-H* loops for $Co_{0.9}Fe_{0.1}$ deposited on $BiFeO_3$ (red), $Bi_{0.85}La_{0.15}FeO_3$ (blue), and $DyScO_3$ (substrate, black), respectively. This indicates that the in-plane exchange coupling between the $Co_{0.9}Fe_{0.1}$ layer and the $BiFeO_3$ layer is becoming smaller with lanthanum substitution.

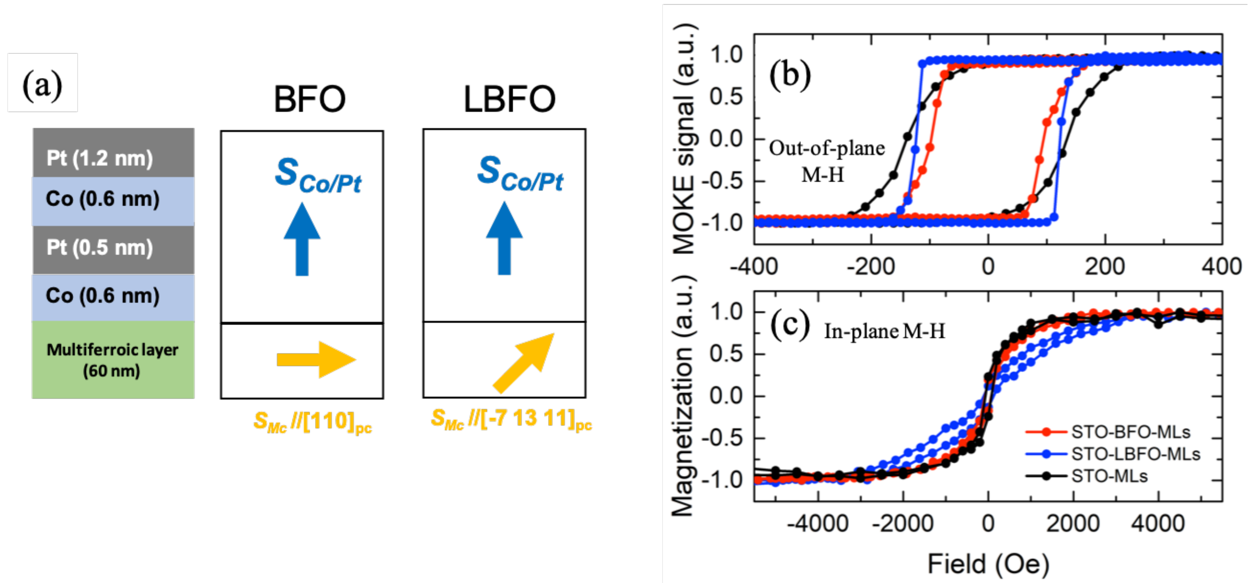

Supplementary Figure 13. Magnetic hysteresis loops of the Co-Pt multilayer/(L) $BiFeO_3$ heterostructures measured along the out-of-plane direction. (a) Schematic of Co-Pt multilayer/ $BiFeO_3$ ($Bi_{0.85}La_{0.15}FeO_3$) heterostructure. Also shown is a schematic description of the exchange coupling between the canted moment, $\mathbf{M_C}$ in the $BiFeO_3$ (or $Bi_{0.85}La_{0.15}FeO_3$) layer and the moment in the ferromagnet, Co/Pt. (b) Out-of-plane M-H loops for $Bi_{0.85}La_{0.15}FeO_3$, $BiFeO_3$, and STO with the same Co-Pt multilayer. (c) In-plane M-H loops for $Bi_{0.85}La_{0.15}FeO_3$, $BiFeO_3$, and STO with the same Co-Pt multilayer. Comparing (b) and (c), it shows that a stronger PMA (perpendicular magnetic anisotropy) can be established on $Bi_{0.85}La_{0.15}FeO_3$ than

$BiFeO_3$. Together with Supplementary Figure 12, the exchange coupling between the ferromagnetic layer and the multiferroic layer tends to be stronger toward the out-of-plane direction with lanthanum substitution in $BiFeO_3$ thin films.

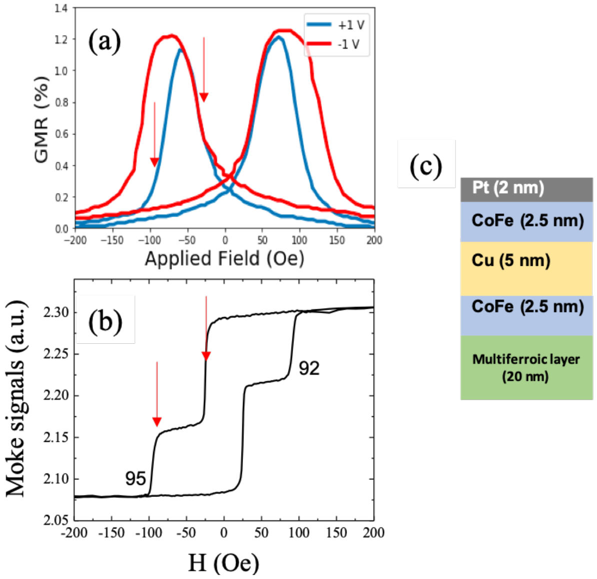

Supplementary Figure 14. (a) Typical R(H) hysteresis of a spin-valve/$Bi_{0.85}La_{0.15}FeO_3$ heterostructure. The arrows indicate the magnetic coercive fields of two $Co_{0.9}Fe_{0.1}$ layers. (b) longitudinal-MOKE (Magneto-optic Kerr effect) hysteresis of a spin-valve/$Bi_{0.85}La_{0.15}FeO_3$ heterostructure. (c) Schematic for a spin-valve/$Bi_{0.85}La_{0.15}FeO_3$ heterostructure.

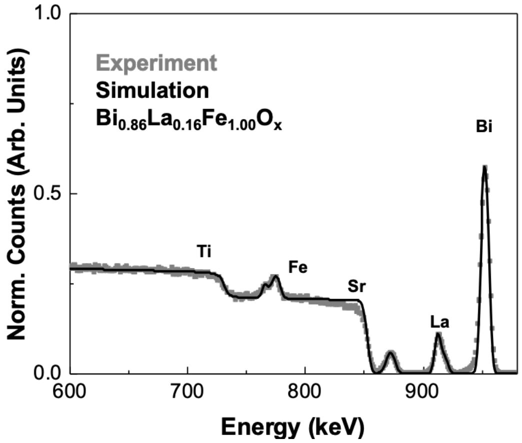

Supplementary Figure 15. Chemical composition measured by Rutherford backscattering spectrometry of a 20-nm-thick $Bi_{0.85}La_{0.15}FeO_3$ sample. We note that this technique typically has 1 atomic percent error for thin-film samples.

Supplementary Reference:

1. Holcomb, M.B. et al. Probing the evolution of antiferromagnetism in multiferroics. *Phys. Rev. B*, **81**, 134406 (2010).

Micromagnet simulations:

The micromagnetic simulations have been performed using the National Institute of Standards and Technology simulator, OOMMF

An example of an OOMMF input script is as follows:

```
# MIF 2.1
# hysteresis of a ferromagnet under exchange bias from BFO
# in-plane magnetization
# rectangular nanomagnet
# stripes of exchange bias
# constants
set pi [expr 4*atan(1.0)]                    ;# number pi
set mu0 [expr 4*$pi*1e-7]                    ;# premeability of vacuum, kg*m/C^2
Parameter Temp 300                           ;# temperature in K
set seed [expr int(714025.0 * rand())]
# material parameters
Parameter Ms 600e3                           ;# free layer magnetization, A/m
Parameter Ms_r 1000e3                        ;# reference magnetization, A/m
Parameter Ax  3e-12                          ;# exchange stiffness, J/m
Parameter alp 0.01                           ;# Gilbert damping
Parameter Lambda 1.0                         ;# angle dependence of spin torque
Parameter K1 0e4                             ;# cubic anisotropy, J/m^3
Parameter Ku_b 0e5                           ;# free layer one axis anisotropy, J/m^3
Parameter Ku_t 0e5                           ;# reference layer one axis anisotropy, J/m^3
Parameter Heb0 0                             ;# Exchange bias field, Oe
set ws 100000e-9                                     ;# width of two stripes, m
Parameter Kec 5000                           ;# exchange coupling energy w BFO, J/m^3
Parameter refcoupl 3e-7                      ;# free to reference layer exchange coupling, J/m^2
set Heb [expr {$Heb0*1e-4/$mu0}]  ;# Convert to A/m, with a factor to make a unit vector
# positive voltage, GMR0
set cec1 [list 0.8242    0.5494   0]         ;# vectors of exchange coupling, main stripe
set cec2 [list 0.8242    0.5494   0]         ;# vectors of exchange coupling, other stripe
set vec1 [list -0.3925    0.7290    0.5608]          ;# vectors of exchange bias, main
stripe
set vec2 [list -0.3925    0.7290    0.5608]       ;        # vectors of exchange bias, other
stripe
# negative voltage, GMR0
#set cec1 [list 0.4472         0   0]                  ;# vectors of exchange coupling,
main stripe
#set cec2 [list 0.4472         0   0]                  ;# vectors of exchange coupling,
other stripe
#set vec1 [list -0.3651   -0.9129   -0.1826]   ;# vectors of exchange bias, main stripe
#set vec2 [list -0.3651   -0.9129   -0.1826]   ;# vectors of exchange bias, other stripe
# geometry
Parameter asp 10                             ;# aspect ratio
Parameter Lnm 200                            ;# feature size, nm
set L [expr 1e-9*$Lnm]                               ;# feature size, m
set width  [expr $L]
set length [expr {$asp*$width}]
set thick    1e-9
```

```
Parameter xycellsize 20e-9
set zcellsize [expr {1*$thick}]
set z1 [expr {1*$thick}]
set z2 [expr {2*$thick+$z1}]
set z3 [expr {$thick+$z2}]
Parameter curad 50e-9                    ;# radius of rounded corners, m
Parameter asprad 2                  ;# aspect ratio of the rounded corner
# device parameters
Parameter Happ 300.0                     ;# External field, Oe
set Happ [expr {$Happ*1e-4/$mu0}]        ;# Convert to A/m
Parameter Hdip 0.0                  ;# Dipole field, Oe
set Hdip [expr {$Hdip*1e-4/$mu0}]        ;# Convert to A/m
Parameter co 0.98                   ;# cosine along main direction
Parameter devx 0.0                  ;# deviation in x
Parameter devy 0.1                  ;# deviation in y
Parameter devz 0.1                  ;# deviation in z
# vector of initial magnetization
Parameter mo_theta 0.0              ;# Direction of mo, in degrees
set mo_theta [expr {$mo_theta*$pi/180.}]
Parameter mo_phi 0.0                     ;# Direction of mo, in degrees
set mo_phi [expr {$mo_phi*$pi/180.}]
set ovect [list [expr {cos($mo_theta)}] [expr {sin($mo_theta)*cos($mo_phi)}] [expr
{sin($mo_theta)*sin($mo_phi)}] ]
# execution options
set basename [subst hy$Lnm ]
Specify Oxs_BoxAtlas:freelay [subst {
  xrange {0 $length}
  yrange {0 $width}
  zrange {0 $z1}
}]
Specify Oxs_BoxAtlas:spacer [subst {
  xrange {0 $length}
  yrange {0 $width}
  zrange {$z1 $z2}
}]
Specify Oxs_BoxAtlas:topref [subst {
  xrange {0 $length}
  yrange {0 $width}
  zrange {$z2 $z3}
}]
Specify Oxs_MultiAtlas:vsyo {
atlas :freelay
atlas :spacer
atlas :topref
}
Specify Oxs_MultiAtlas:magnets {
```

```
atlas :freelay
atlas :topref
}
Specify Oxs_RectangularMesh:mesh [subst {
  cellsize {$xycellsize $xycellsize $zcellsize}
  atlas :vsyo
}]
# Geometry of the output electrode
Specify Oxs_ScriptScalarField:OutputEl [subst {
            atlas :vsyo
            script {Poln 1}
            script_args relpt
}]
Specify Oxs_AtlasScalarField:OutputEl_f [subst {
            atlas :vsyo
            default_value 0
            values {
                        freelay :OutputEl
            }
}]
Specify Oxs_AtlasScalarField:OutputEl_r [subst {
            atlas :vsyo
            default_value 0
            values {
                        topref :OutputEl
            }
}]
Specify Oxs_AtlasScalarField:Ktot [subst {
    atlas :magnets
    default_value 0
    values {
        freelay $K1
                        topref $K1
            }
}]
# Cubic anisotropy
Specify Oxs_CubicAnisotropy [subst {
  K1  :Ktot
  axis1 {1 0 0}
  axis2 {0 1 0}
}]
Specify Oxs_AtlasScalarField:Kunitot [subst {
    atlas :magnets
    default_value 0
    values {
        freelay $Ku_b
```

```
                    topref $Ku_t
          }
}]
# Uniaxial anistropy.
Specify Oxs_UniaxialAnisotropy:pma [subst {
  K1 :Kunitot
  axis {0 0 1}
}]
Specify Oxs_Exchange6Ngbr [subst {
default_A 0.0
atlas magnets
A {
freelay freelay $Ax
topref topref $Ax
}
}]
# Demag
Specify Oxs_Demag {}

Specify Oxs_ScriptVectorField:stripeEB [subst {
          script {StripesLim $ws $vec1 $vec2}
          script_args {relpt span}
          atlas :freelay
}]
# exchange bias field
Specify Oxs_FixedZeeman:exchbias [subst {
   field :stripeEB
   multiplier $Heb
}]
Specify Oxs_ScriptVectorField:stripeEC [subst {
          script {Stripes $ws $cec1 $cec2}
          script_args {relpt span}
          atlas :freelay
}]
Specify Oxs_AtlasScalarField:Kectot [subst {
   atlas :magnets
   default_value 0
   values {
      freelay $Kec
          }
}]
Specify Oxs_UniaxialAnisotropy:coeenh [subst {
  K1 :Kectot
  axis :stripeEC
}]
# field with a slight angle to x
```

```
# { 0 0 0 [expr {$Happ*$co}] [expr {$Happ*$devy} ]  [expr {$Happ*$devz}]   100 }
Specify Oxs_UZeeman [subst {
  Hrange {
{ [expr {$Happ*$co}] [expr {$Happ*$devy}] [expr {$Happ*$devz}]  [expr {-$Happ*$co}]
[expr {-$Happ*$devy}]  [expr {-$Happ*$devz}]   200 }
{ [expr {-$Happ*$co}] [expr {-$Happ*$devy}] [expr {-$Happ*$devz}] [expr {$Happ*$co}]
[expr {$Happ*$devy} ]  [expr {$Happ*$devz}]   200 }
  }
}]
# Magnetization
Specify Oxs_ScriptScalarField:Ms [subst {
        script {RoundedCorners $Ms $curad $asprad}
        script_args {relpt span}
        atlas :freelay
}]
Specify Oxs_ScriptScalarField:Msr [subst {
        script {RoundedCorners $Ms_r $curad $asprad}
        script_args {relpt span}
        atlas :topref
}]
Specify Oxs_AtlasScalarField:Mstot [subst {
    atlas :vsyo
    default_value 0
    values {
       freelay :Ms
              topref :Msr
        }
}]
Specify Oxs_AtlasScalarField:alp [subst {
    atlas :vsyo
    default_value 1
    values {
       freelay $alp
              topref $alp
        }
}]
Specify Oxs_LinearScalarField:zheight {
vector {0 0 1}
norm 1.0
}
Specify Oxs_TwoSurfaceExchange:FMexc [subst {
sigma $refcoupl
surface1 {
atlas :vsyo
region freelay
scalarfield :zheight
```

```
scalarvalue $z1
scalarside -
}
surface2 {
atlas :vsyo
region topref
scalarfield :zheight
scalarvalue $z2
scalarside +
}
}]
# initial state
Parameter Input ""
if { [string length $Input] > 0 } {
   # we'll assume that readability has been checked externally
   Specify Oxs_FileVectorField:init [ subst {
         file $Input
         atlas :magnets
   } ]
} else {
         Specify Oxs_AtlasVectorField:init [ subst {
         atlas :magnets
         norm 1
         default_value {0. 0. 0.}
         values {
                  freelay {[lindex $ovect 0] [lindex $ovect 1] [lindex $ovect 2]}
                  topref {[lindex $ovect 0] [lindex $ovect 1] [lindex $ovect 2]}
                  }
         } ]
}
# projection fields for output
Specify Oxs_MaskVectorField:mxout_f {
         field {1 0 0}
         mask :OutputEl_f
}
Specify Oxs_MaskVectorField:myout_f {
         field {0 1 0}
         mask :OutputEl_f
}
Specify Oxs_MaskVectorField:mzout_f {
         field {0 0 1}
         mask :OutputEl_f
}
Specify Oxs_MaskVectorField:mxout_r {
         field {1 0 0}
         mask :OutputEl_r
```

```
}
Specify Oxs_MaskVectorField:myout_r {
        field {0 1 0}
        mask :OutputEl_r
}
Specify Oxs_MaskVectorField:mzout_r {
        field {0 0 1}
        mask :OutputEl_r
}
# start the simulation
   # Evolver
   Specify Oxs_CGEvolve:evolve {}
   # Driver
   Specify Oxs_MinDriver [subst {
                basename [list $basename]
                evolver :evolve
                stopping_mxHxm 0.1
                checkpoint_interval 5
                stage_iteration_limit 1000
                mesh :mesh
                Ms :Mstot
                m0 :init
                projection_outputs {
                Mfx :mxout_f
                Mfy :myout_f
                Mfz :mzout_f
                Mrx :mxout_r
                Mry :myout_r
                Mrz :mzout_r
                }
   }]
# end the simulation
proc Poln { Ms x y z } {
   if {$x<0 || $x>1 || $y<0 || $y>1} {return 0.0}
   return $Ms
}
proc RoundedCorners { Ms curad asprad x y z xspan yspan zspan} {
   set xoff [expr {$x*$xspan-$xspan+$curad*$asprad}]
   set yoff [expr {$y*$yspan-$yspan+$curad}]
   if {abs($xoff)*$xoff/$asprad/$asprad+abs($yoff)*$yoff>$curad*$curad} {return 0.0}
   set xoff [expr {$x*$xspan-$xspan+$curad*$asprad}]
   set yoff [expr {-$y*$yspan+$curad}]
   if {abs($xoff)*$xoff/$asprad/$asprad+abs($yoff)*$yoff>$curad*$curad} {return 0.0}
   set xoff [expr {-$x*$xspan+$curad*$asprad}]
   set yoff [expr {$y*$yspan-$yspan+$curad}]
   if {abs($xoff)*$xoff/$asprad/$asprad+abs($yoff)*$yoff>$curad*$curad} {return 0.0}
```

```
   set xoff [expr {-$x*$xspan+$curad*$asprad}]
   set yoff [expr {-$y*$yspan+$curad}]
   if {abs($xoff)*$xoff/$asprad/$asprad+abs($yoff)*$yoff>$curad*$curad} {return 0.0}
   if {$x<0 || $x>1 || $y<0 || $y>1} {return 0.0}
   return $Ms
}
proc Stripes {ws v1x v1y v1z v2x v2y v2z x y z xspan yspan zspan} {
         set xoff [expr {$x*$xspan}]
   set yoff [expr {($y-0.)*$yspan}]
   set dialin [expr {$xoff+$yoff+0*$ws}]
   set strdef [expr {fmod($dialin,$ws)/$ws}]
   if {$strdef<0.5} {return [list $v1x $v1y $v1z ]} else {return [list $v2x $v2y $v2z]}
}
proc StripesLim {ws v1x v1y v1z v2x v2y v2z x y z xspan yspan zspan} {
         if {$z<0 || $z>1} {return [list 0.0 0.0 0.0] }
         set xoff [expr {$x*$xspan}]
   set yoff [expr {($y-0.)*$yspan}]
   set dialin [expr {$xoff+$yoff+0*$ws}]
   set strdef [expr {fmod($dialin,$ws)/$ws}]
   if {$strdef<0.5} {return [list $v1x $v1y $v1z ]} else {return [list $v2x $v2y $v2z]}
}
# specifications for data outputs
Destination archive mmArchive
Schedule DataTable archive Stage 1
Destination datatab mmDataTable
Schedule DataTable datatab Stage 1
Destination graph mmGraph
Schedule DataTable graph Stage 1
#Schedule Oxs_MinDriver::Magnetization archive Stage 1
Destination display mmDisp
Schedule Oxs_MinDriver::Magnetization display Stage 5
```
